# Essential oil of tarragon (*Artemisia dracunculus*) antibacterial activity on *Staphylococcus aureus* and *Escherichia coli* in culture media and Iranian white cheese

**Published:** 2012-03

**Authors:** M Raeisi, H Tajik, Roohani SM Razavi, M Maham, M Moradi, B Hajimohammadi, H Naghili, M Hashemi, T Mehdizadeh

**Affiliations:** 1 Department of Food Quality Control, Faculty of Veterinary Medicine, Urmia university, Urmia, Iran; 2 Department of Clinical Sciences, Faculty of Veterinary Medicine, Urmia university,Urmia, Iran; 3 Department of Food Science and Technology, Ahar Faculty of Agriculture, University of Tabriz, Tabriz, Iran; 4 Department of Food Hygiene and Safety, Faculty of Health, Shahid Sadoughi University of Medical Sciences, Yazd, Iran

**Keywords:** Tarragon, essential oil, antibacterial effect, *Staphylococcus aureus*, *Escherichia coli*, cheese

## Abstract

**Background and Objectives:**

In this study, the antibacterial effect of essential oil of tarragon (*Artemisia dracunculus*) on *Staphylococcus aureus* and *Escherichia coli* was evaluated in culture media and Iranian white cheese.

**Materials and Methods:**

The tarragon essential oil (EO) obtained by the steam distillation method and its antibacterial activity was evaluated in 96-well microtiter plates containing brain heart infusion broth. The enumeration of *S. aureus* and *E. coli* in cheese samples were carried out on the following media: Baired parker agar for *S.aureus*, incubated at 37°C for 24 h; and MacConkey sorbitol agar for *E. coli*, incubated at 37°C for 24 h. Iranian white cheese was produced from fresh and whole pasteurized cow milk (2.5%). Bacteria (10^3^ cfu/mL) were inoculated to different batches. Cheese was treated with different concentrations of EO (15 and 1500 µg/mL) and separated into four parts in an equal manner. The sensory evaluation was done by a panel of four judges.

**Results:**

According to the results obtained, minimum inhibitory concentrations (MIC) for *E. coli* and *S. aureus* were 2500 and 1250 µg/mL, respectively. Also, minimum bactericidal concentration (MBC) for the mentioned microorganisms were 5000 and 2500 µg/mL, respectively. All the EO concentrations for each bacteria result in reducing bacterial count of cheese samples compared to control (*P*<0.05). Also, with increasing concentration of EO in cheese samples, the bacterial count was reduced further (*P*<0.05).

**Conclusion:**

Based on our findings, tarragon essential oil has antibacterial effect on two important pathogen bacteria (*S. aureus and E. coli*) and can be applied as a preservative in foods such as cheese.

## INTRODUCTION

Nowadays, foods protected with natural additives have been well-liked due to increasing awareness and concern of consumers about synthetic chemical preservatives. Plant essential oils are from natural chemical sources used for preserving food. On the other hands, there still exists a very important concern about increasing of food borne diseases caused by some pathogens such as *Staphylococcus aureus* and *Escherichia coli*. It was approved that some plant essential oils have powerful antibacterial ingredients against food borne pathogens (1–6).

Tarragon (*Artemisia dracunculus* L., *Asteraceae* family) is a plant having several diverse common names such as estragon, dragon sage-wort, false tarragon and dragon wormwood. The plant is cultivated in many countries including Iran. The main compound of the essential oil of this plant is methylchavicol (estragol) (7).

Based on our knowledge, although some researchers have worked on the antibacterial effects of essential oil of tarragon against some pathogenic microorganisms in culture media (8–10), a comparative study in both culture media and a food model system (e.g. cheese) has not been published. The main objective of this study was to evaluate the antibacterial effect of essential oil of tarragon on *S. aureus* and *E. coli* in culture media and cheese.

## MATERIALS AND METHODS

### Preparation of essential oil

The plant was purchased from the local grocery of Urmia city and the essential oil (EO) of the plant was extracted by steam distillation and dried by adding anhydrous sodium sulphate, filter sterilized by 0.22-mm filter and stored at 4°C before being used for assay.

### Microorganisms

The *S. aureus* ATCC 6538 and *E. coli* PTCC 1533 were obtained kindly from Department of Microbiology, Faculty of Veterinary Medicine, University of Urmia, Urmia, Iran.

### Preparation of inocula

*S.aureus* and *E. coli* inocula were produced by transferring bacteria from working culture to Brain heart infusion (BHI) broth and then incubating at 37°C for 18–22 h. After second subculture, bacteria were incubated for 20 h at 37°C. The bacterial cultures were fitted to optical density by using a spectrophotometer (600 nm) to acquire a bacterial concentration of 10^6^ cfu/mL. Bacterial counts were verified by plating the different dilutions on BHI agar and incubated at 37°C for 24 h (11).

### Determination of MIC and MBC on culture media

Stock solution of tarragon EO (100000 µg/mL) in 10% dimethylsulfoxide (DMSO) was prepared. Then, two fold serial dilutions of EO (97.5, 195, 390, 781, 1562, 3125, 6250, 12500, 25000 and 50000 µg/mL) were prepared. At first, 160 µL of sterile broth was added to each well of a 96-well microtiter-plate. Then, 20 µL of the microbial suspension and 20 µL of each EO concentrations were added to the designed wells. Thus, the achieved EO concentrations were 19.5, 39, 78.1, 156.2, 312.5, 625, 1250, 2500 and 5000 µg/mL. For every experiment, two growth controls consisting of BHI broth without essential oil and BHI broth containing DMSO inoculated with the diluted medium culture and one sterility control containing essential oil were run in each plate and plates were finally incubated at 37°C for 24 h. The MICs were chosen as the least concentrations of the EO resulting in perfect inhibition of visible growth in the broth medium.

To evaluate the MBCs of the EO, 0.1 mL from nonturbid wells were subcultured on BHI agar and incubated at 37°C for 24 h. Then, the lowest concentrations of EO that allowed less than 0.1% of the original inoculum to survive was considered as MBCs (12).

### Determination of the effects of tarragon EO on inoculated cheese

Iranian white cheese was produced from fresh and whole pasteurized cow milk (2.5%). Bacteria (10^3^ cfu/mL) were inoculated in different batches. Then, the rennet (0.001%) and EO were added and maintained at 35°C. For *E. coli*, the amount of EO were 2500 and 3500 µg/mL and for *S. aureus* were 1500 and 2500 µg/mL. One control group was designed without EO.

For evaluation of the effects of EO on inoculated microorganisms, bacterial counts were performed by plating out on plate agar, in four intervals (0, 24, 48 and 72 h).

Decimal dilutions (1:10) in 0.1% peptone water solution were prepared. The enumeration of *S. aureus* and *E. coli* in cheese samples were carried out on the following media: Baired parker agar for *S.aureus*, incubated at 37°C for 24 h; and MacConkey sorbitol agar for *E. coli*, incubated at 37°C for 24 h.

### Sensory evaluation

The cheese treated with different concentrations of EO (15 and 1500 µg/mL) and separated into four parts in an equal manner. The sensory evaluation was done by a panel of four judges, consisting of staffs of the Department of Food Hygiene and Quality Control, Faculty of Veterinary Medicine, University of Urmia. Each member judged the cheese samples for different characteristics including flavor and odor (11).

Nine-point scale was considered containing: 9 = like extremely, 8 = like very much, 7 = like moderately, 6 = like slightly, 5 = neither like nor dislike, 4 = dislike slightly, 3 = dislike moderately, 2 = dislike very much and 1 = dislike extremely.

For evaluation of sensory effect of tarragon essential oil, control model prepared without essential oil, mentioned as number 9 in scale system.

### Statistical analysis

All experiments were done in triplicate. Statistical analysis was performed using SPSS software. The results showing p < 0.05 were considered as significant.

## RESULTS

### MICs and MBCs of the bacteria on culture media

The results obtained from three repetitions for each bacterium were similar. MICs for *E. coli* and *S. aureus* were 2500 and 1250 µg/mL, respectively. Also, MBCs for the mentioned bacteria were 5000 and 2500 µg/mL respectively. So, according to these findings, *S. aureus* was more sensitive than *E. coli* to tarragon EO.

### Effect of EO on the bacteria inoculated to cheese

**Fig. 1:**
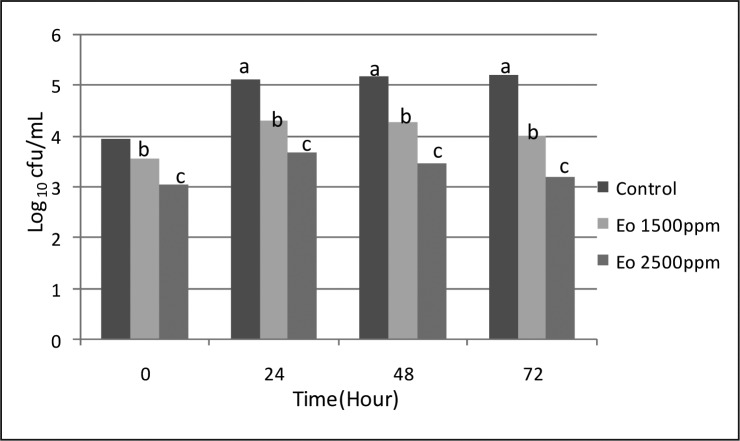
*S. aureus* count of cheese samples (log10 cfu/mL) in different intervals and different concentration of tarragon essential oil (different letters have significant difference (p < 0.05)).

**Fig. 2:**
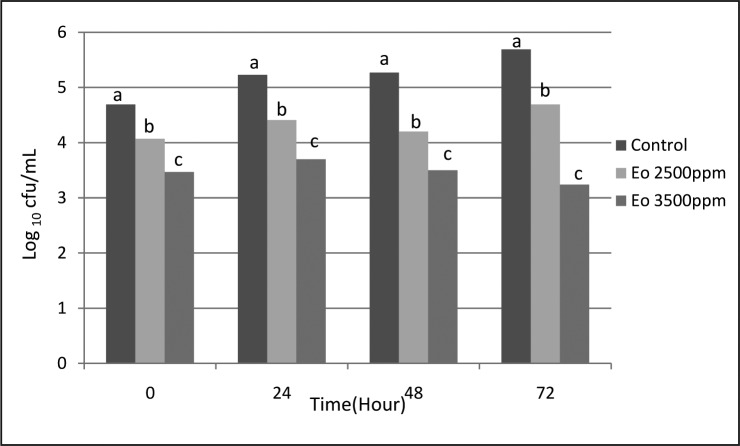
*E. coli* count of cheese samples (log10 cfu/mL) in different intervals and different concentration of tarragon essential oil (different letters have significant difference (p < 0.05)).

The results obtained for *S. aureus* and *E. coli* are shown in Figs. 1 & 2, respectively. In all EO concentrations, bacterial counts were reduced compared to the control group (*P*<0.05). With increasing concentration, the bacterial count was significantly decreased (*P*<0.05), whereas, any significant difference between different intervals (0, 24, 48 and 72 h) were not achieved (*P*>0.05).

### Sensory evaluation

**Fig. 3:**
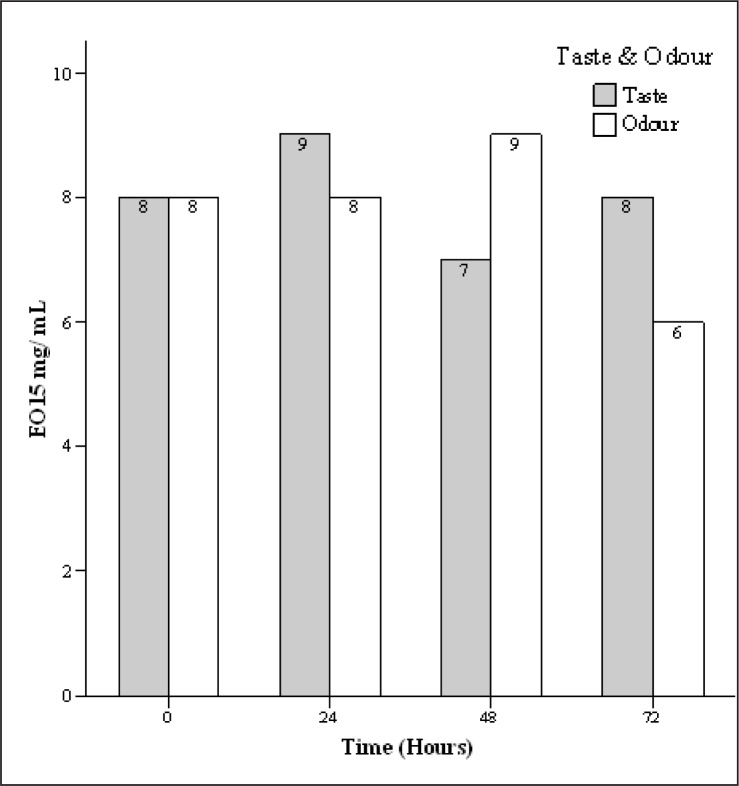
Sensory effect of 15 µg/mL tarragon essential oil (EO) concentration in different intervals.

**Fig. 4:**
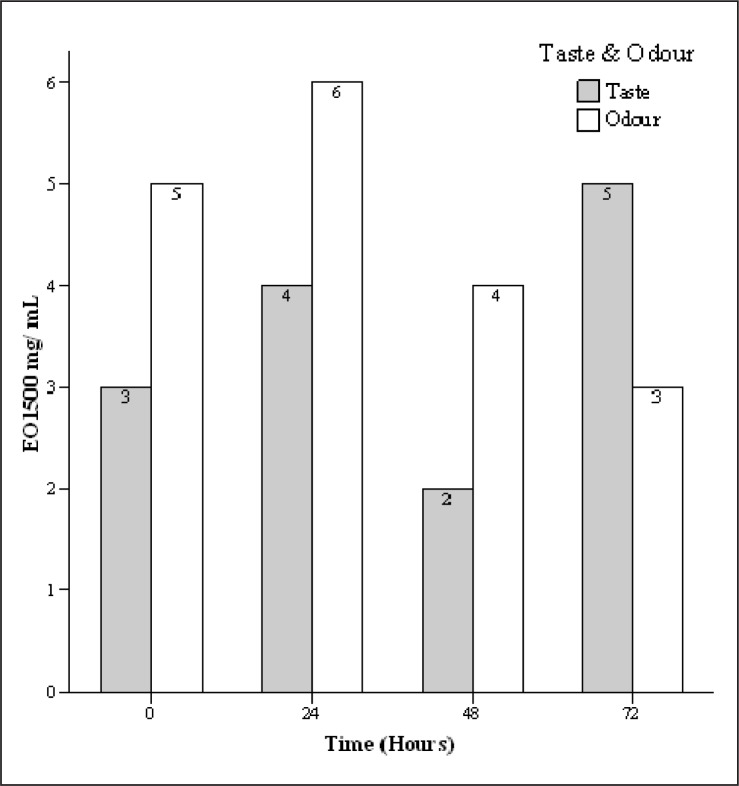
Sensory effect of 1500 µg/mL tarragon essential oil (Eo) concentration in different intervals.

The mean acceptability score of cheese samples containing 15 µg/mL of EO was 8 for taste and 7.7 for odour characteristic and considered suitable (Fig. 3). Also, the mean acceptability score of cheese samples containing 1500 µg/mL of EO was 3.5 for taste and 4.5 for odour characteristic and not considered favorable (Fig. 4).

## DISCUSSION

It is clearly demonstrated that some plant essential oils possesses antibacterial compounds. In spite of this, comparing the results of different studies is not easy due to differences in assay methods, type of bacteria and the origin and geographical properties of the plant (4, 13, 14).

In the present study, MICs for *E. coli* and *S. aureus* were 2500 and 1250 µg/mL, respectively. Also, MBC for the mentioned bacteria were 5000 and 2500 µg/mL respectively. So, the results revealed that *S. aureus* is more sensitive than *E. coli* to tarragon EO. A similar study performed by Bonyadian and Karim (2002) reported that the MIC and MBC of tarragon against *E. coli* were 4% and 5% respectively. Also, those rates for *S. aureus* were 5% and 7% respectively (8). In another research, the significant antibacterial effect of methanol extract of tarragon on *E. coli* had been investigated (15). Bonyadian and Moshtaghi (2008) evaluated the effect of tarragon EO on four pathogenic bacteria including *Salmonella Typhimurium*, *Listeria monocytogenes*,*Yersinia enterocolitica* and *bacillus cereus*. Among the mentioned bacteria, the most sensitive and resistant bacteria were *Y. enterocolitica* and *S*. *Typhimurium*, respectively (9). In a similar study, the MIC and MBC of tarragon EO for *S. aureus* were 7% and 9% respectively and for *E. coli* were 6% and 8% respectively (10).

In general, Gram-negative bacteria are more resistant to plant EO than Gram-positives (4) which is in accordance with our findings. This phenomenon is attributed to the presence of an outer membrane in Gram-negatives, which have hydrophilic polysaccharide chains acting as an obstacle to hydrophobic essential oils (16).

In spite of the powerful antibacterial effects of essential oils on food borne microorganisms, their practical use is restricted due to their displeasing flavor change in foods. Moreover, their effectiveness in food is less than culture media (4, 11, 13). Based on our work, the amount of tarragon EO required to make antibacterial activity in cheese was more than the amount used commonly as favoring and so made displeasing sensorial effects. Thus, it is suggested that tarragon EO be used as a part of a combination with other preservation methods to reduce its concentrations and consequent unfavorable sensorial effects (13).

In general, it is concluded that tarragon EO has an antibacterial effect on two important pathogen bacteria (*S. aureus and E. coli*) and can be applied as a natural preservative in food such as cheese. However, more studies are required to determine the economical costs and practical use of this plant EO in commercial settings.

## References

[1] Blackburn CW, McClure PJ (2009). Foodborne pathogens.

[2] Friedman M, Henika PR, Mandrell RE (2002). Bactericidal activities of plant essential oils and some of their isolated constituents against *Campylobacter jejuni, Escherichia coli, Listeria monocytogenes*, and *Salmonella enterica*. J Food Prot.

[3] Holley RA, Patel D (2005). Improvement in shelf-life and safety of perishable foods by plant essential oils and smoke antimicrobials. Food Microbiology.

[4] Jay JM, Loessner MJ, Golden DA (2005). Modern food microbiology.

[5] Montville TJ, Matthews KR (2008). Food microbiology.

[6] Nedorostova L, Kloucek P, Kokoska L, Stolcova M, Pulkrabek J (2009). Antimicrobial properties of selected essential oils in vapour phase against food borne bacteria. Food Control.

[7] Aglarova AM, Zilfikarov IN, Severtseva O.V (2008). Biological characteristics and useful properties of tarragon (*Artemisia dracunculus*). Pharmaceutical Chemistry J.

[8] Bonyadian M, Karim G (2002). Study of the effects of some volatile oils of herbs against *Escherichia coli* and *Staphylococcus aureus* in broth media. J Fac Vet Med Univ Tehran.

[9] Bonyadian M, Moshtaghi H (2008). Bactericidal activity of some plants essential oils against *Bacillus cereus*, *Salmonella typhimurium*, *Listeria monocytogenes* and *Yersinia enterocolitica*. Res J Microbiol.

[10] Mohsenzadeh M (2007). Evaluation of antibacterial activity of selected Iranian essential oils against *Staphylococcus aureus* and *Escherichia coli* in nutrient broth medium. Pak J Bio Scie.

[11] Moosavy M, Basti A, Misaghi A, Salehi T, Abbasifar R, Mousavi HA (2008). Effect of *Zataria multiflora* Boiss. essential oil and nisin on *Salmonella typhimurium* and *Staphylococcus aureus* in a food model system and on the bacterial cell membranes. Food Research International.

[12] Baron EJ, Peterson LR, Finegold SM (1994). Diagnostic Microbiology.

[13] Misaghi A, Basti A (2007). Effects of *Zataria multiflora* Boiss. essential oil and nisin on *Bacillus cereus* ATCC 11778. Food Control.

[14] Naidu AS (2000). Natural food antimicrobial systems.

[15] Benli M, Kaya I, Yigit N (2007). Screening antimicrobial activity of various extracts of *Artemisia dracunculus* L. Cell Biochem Funct.

[16] Amor IL, Neffati A, Sgaier MB, Bhouri W, Boubaker J, Skandrani I (2008). Antimicrobial Activity of Essential Oils Isolated from Phlomis *crinita* Cav. ssp. *mauritanica* Munby. J Am Oil Chem Soc.

